# Role of pattern recognition receptors and microbiota-derived ligands in obesity

**DOI:** 10.3389/frmbi.2024.1324476

**Published:** 2024-03-08

**Authors:** Alice Rolland, Véronique Douard, Nicolas Lapaque

**Affiliations:** Paris-Saclay University, INRAE, AgroParisTech, Micalis Institute, Jouy-en-Josas, France

**Keywords:** microbiota, PRRs, MAMPs, obesity, TLR, NLRP3, NOD

## Abstract

Obesity is associated with activation of low-grade inflammation in tissues metabolically relevant for the regulation glucose homeostasis. The gut microbiota has been extensively linked to the inflammatory responses observed during obesity emphasizing the interconnection between host immunity and metabolism during obesity. Gut microbiota together with alteration of the gut barrier functions provide a myriad of circulating ligands for the pattern recognition receptors (PRRs) expressed in innate immune cells and nonimmune cells. PRR-dependent signalling drives the expression of a wide range of genes beyond the inflammatory response depending on the specific functions of the targeted cells and on the physiological context. PRRs activation can have opposite effects on host metabolic inflammation. Nucleotide-binding oligomerization domain 1 (NOD1) or NOD-like Receptor pyrin domain containing 3 (NLRP3) activation promote metabolic inflammation and insulin resistance while NOD2 activation improves insulin sensitivity and glucose homeostasis during obesity. Toll-like receptors (TLRs) 2, 4 and 5 also display specific effects on metabolic tissues. TLR5 deficient mice are prone to obesity and inflammation in response to high fat diet, while injection of TLR5 ligand, flagellin, has a protective effect toward diet-induced obesity. To the opposite TLR2 and 4 activations are associated with deleterious metabolic outcome during obesity. TLR4 activation enhances metabolic inflammation and insulin resistance and TLR2 via its activation by molecules derived from the gut microbiota favours the onset of obesity. It is now clear that activation of PRRs by bacterial derived molecules plays a key role in the host metabolic regulation. PRRs are expressed in various cell types complicating the understanding of the mechanisms underlying the relationship between PRRs activation/silencing and metabolic inflammation in obesity context. This review presents an overview of the current understanding of the interrelationship between the gut microbiota and PRRs, with a focus on its consequences for obesity and related metabolic diseases.

## Introduction

Over the past decades, overweight and obesity have reached epidemic proportions worldwide, affecting around 1.9 billion and 650 million people, respectively in 2016 i.e 13% of the world’s adult population, as per World Health Organization (WHO, 2018). The worldwide prevalence of obesity has nearly tripled since 1975, and the World Obesity Federation estimated that over one billion people worldwide would be living with the disease in 2030 (WHO, 2018).

Obesity is associated with metabolic disorders such as cardiovascular diseases and type 2 diabetes, with chronic diseases such as Non Alcoholic Fatty Liver (NAFL), musculoskeletal diseases and chronic kidney disease, and predisposes to certain types of cancers and autoimmune diseases (Swinburn et al., 2011). Obesity and overweight are responsible for 2.8 million deaths per year worldwide and their associated comorbidities negatively affect subjects’ quality of life and have important implications for public health and healthcare systems. The recent COVID-19 pandemic profoundly impacted obesity and overweight evolution and outcomes. Indeed, early studies indicate that the prevalence of overweight and obesity has increased in children and adolescents during the COVID-19 pandemic due to unfavourable shifts in food consumption and decrease in physical activity (Stefan et al., 2021). Moreover, obese patients have been more affected by the COVID-19 pandemic consequences, due to complications from the viral infection and disruptions in the access of their obesity management. Obesity is therefore considered to be one of the most important health problems of both westernized and developing countries (WHO, 2018). Severe obesity is almost incurable. Bariatric surgery has been considered the most effective strategy for losing weight and treating obesity-related comorbidities. However, such surgical procedure affects the gut anatomy and main functions (motility and nutrient digestion and absorption). Therefore, it is associated with many side effects that have deleterious consequences on the health of the patient. Thus, development of new therapeutic approaches is needed and will require a broad understanding of the causes of obesity and the associated metabolic diseases.

Obesity is a multifactorial disease, involving genetic, physiological, metabolic, behavioural, social and environmental factors that can all be related to low-grade systemic chronic inflammation [for review see (Calder et al., 2011; Calder, 2022)]. These factors include sedentary lifestyle with alterations in the intestinal microbiota colonization (e.g. antibiotics, “western” diet) that emerged in developed societies over the last century. This narrative review aims to highlight the association between inflammation, gut microbiota, innate immune responses and obesity. We will more specifically focus on the microbiota-host crosstalk involving pattern recognition receptors (PRRs) and their ligands, the microbial associated molecular patterns (MAMPs), and its impact on host metabolism.

## Obesity, an inflammatory disease

In the last few decades, it has become clear that the pathophysiology of obesity was associated with chronic subacute inflammation known as low-grade inflammation of the intestine and metabolic tissues, including white and brown adipose tissues, pancreas, hypothalamus and the liver, thereby contributing to insulin resistance and glucose deregulation in animal models (Rehman et al., 2021). First observation of tissue inflammation during obesity showed that high tumour necrosis factor (TNFα) expression in white adipose tissue in obese rodent models was associated with insulin resistance that could be reversed by TNFα neutralization or deletion (Hotamisligil et al., 1993; Uysal et al., 1997). TNF-receptor (TNF-R) stimulation activates multiple signalling pathways such as c-Jun N-terminal kinases (JNK) and the transcription factor nuclear factor kappa B (NF-κB) pathways that are involved in insulin resistance by directly inhibiting the insulin signalling or by inducing the expression of metabolic- and inflammation-related genes (Werner et al., 2004; Baker et al., 2011). Pioneering studies showed that macrophages that are accumulating in adipose tissues during obesity are the main source of TNFα (Weisberg et al., 2003). These observations have been followed by studies demonstrating that adipose tissue macrophages (ATM) can be polarized into pro-inflammatory M1-like and anti-inflammatory M2-like macrophage subtypes and that M1 macrophage play a key role in insulin resistance pathogenesis (Chatzigeorgiou and Chavakis, 2016), even if recent evidence showed that there are multiple nuances in ATM phenotypes adaptation in the context of obesity (Caslin et al., 2020). The liver is also a key organ involved in the response to high-fat diet (HFD)-induced inflammation and insulin resistance (Schuster et al., 2018). In non-alcoholic fatty liver (NAFL), inflammation triggers the progression toward non-alcoholic steatohepatitis (NASH) and hepatic immune cells contribute also to low-grade inflammation associated with obesity (Schuster et al., 2018). In the liver, nearly half of non-hepatocyte cells are immune cells comprising mainly macrophages (Kupffer cells) and lymphocytes including natural killer (NK), NKT and T cells (Crispe, 2009). Under steatosis condition, the Kupffer or NKT cells, loaded with lipids, shift to a pro-inflammatory profile, and the number of regulatory T-cells (Tregs) decreases while lymphocytes recruitment increases (Crispe, 2009). These changes in immune cells profiles, numbers and activation contribute to the development of NASH, liver insulin resistance and inflammation. Diet-induced or genetically inherited obesity are also associated with increased inflammation in the pancreas (Eguchi and Nagai, 2017). This inflammation is characterized by islet immune cell accumulation dominated by local expansion of resident intra-islet macrophages, which ultimately impair β cell functions (Ying et al., 2019).

Altogether these studies established the concept that low-grade chronic inflammation occurs in metabolic tissues (termed metabolic inflammation) and paved the way of many publications showing that adipose tissue, liver or pancreas contain a wide range of immune cell types including lymphocytes, dendritic cells, neutrophils, innate lymphoid cells and natural killer cells involved in the metabolic states of the host (Chatzigeorgiou et al., 2012; Talukdar et al., 2012; Brestoff and Artis, 2015; Chatzigeorgiou and Chavakis, 2016; Lee et al., 2016; O’Sullivan et al., 2016).

## The relationship between gut microbiota and obesity

Recent discoveries have placed the gut microbiota at the forefront of obesity-centred research. Indeed, deep-sequencing approaches of commensal bacterial genomes (metagenome) or bacterial 16S RNA from healthy individuals and obese patients revealed correlations between microbiota composition and, obesity and metabolic disorders (Ley et al., 2005, 2006; Turnbaugh et al., 2006; Le Chatelier et al., 2013). Humans suffering from obesity display a significant alteration of gut microbiota, named dysbiosis, characterized by the reduction in bacterial diversity as well as overall shifts in the composition of bacterial populations (Ley et al., 2006; Turnbaugh et al., 2006; Turnbaugh and Gordon, 2009; Turnbaugh et al., 2009). A large human cohort study showed that obesity, insulin resistance, fatty liver and low-grade inflammation were more prevalent for individuals with low bacterial richness (measured by low gene count) compared to high gene count subjects (Le Chatelier et al., 2013). However, considering the large number of studies that investigated the human faecal microbial composition, few findings on the identification of specific obesity-associated bacteria were consistent. A recent meta-analysis integrating 16S RNA data from 4282 faecal samples confirmed the higher Bacteroidetes/Firmicutes ratio in obese patients as previously reported by some studies (Gong et al., 2022). This meta-analysis also reported a significantly lower abundance of 23 genera confirming previous association of the high abundance of *Christensenellaceae*, *Akkermansia*, *Alistipes* within individuals with body mass index (BMI) inferior to 25 (Goodrich et al., 2014). Metagenomic studies have been applied to hundreds of human faecal samples leading to the classification of microbiota composition into four microbiota profile clusters, also known as enterotypes (Vandeputte et al., 2017). The prevalence of the enterotype Bacteroides2, characterized by high Bacteroides, low *Faecalibacterium* and low microbial cell density, has been shown to correlate with systemic inflammation and increased BMI (Vieira-Silva et al., 2020). Numerous studies in obese and in Type 2 diabetes patients consistently showed a depletion of butyrate-producing bacteria including depletion of *Faecalibacterium*, *Clostridium*, *Alistipes*, and *Roseburia* (Karlsson et al., 2013; Le Chatelier et al., 2013; Forslund et al., 2015; Wu et al., 2020). Many experiments in animal models confirm that alterations of intestinal microbiota are associated with the development of obesity and metabolic disorders (for review see (Ridaura et al., 2013; Cani, 2018). Germ-free mice have been used to determine the role of microbiota on host physiology. Initial studies showed that germ-free animals (animals devoid of microbiota) fed with a regular chow diet have reduced body weight and adiposity compared with conventionally raised animals (Backhed et al., 2004). The most definitive findings that implicated gut microbiota in host metabolism come from pioneering “conventionalization” studies which showed that transfers of the gut microbiota from either lean or obese mice to germ-free mice recapitulate the donor phenotypes in the recipient mice (Turnbaugh et al., 2008). As described above, using 16S RNA, metagenomics and clinical data from cohorts of overweight, obese and lean individuals, many research groups succeeded in identifying several bacterial species negatively or positively associated with the metabolic health. Based on this observational and associative evidence specific taxa were classified as potentially detrimental (negatively associated) or potentially beneficial (positively associated) for the host metabolic health. Furthermore, a number of groups demonstrated a causal effect of specific microbial species or microbial compounds on obesity and related metabolic diseases (Everard et al., 2013; Fei and Zhao, 2013; Plovier et al., 2017; Natividad et al., 2018a, b; Agus et al., 2021; Le Roy et al., 2022). Indeed, colonization of germ-free mice with *Enterobacter cloacae*, *Clostridium ramosum* and *Lachnospiraceae* AJ110941 increased obesity traits (Fei and Zhao, 2013; Kameyama and Itoh, 2014; Woting et al., 2014). Similarly, *Bilophila wadsworthia* aggravated host metabolic dysfunctions induced by westernised diet in conventional mice (Natividad et al., 2018b). In contrast, positively-associated bacteria such as *Akkermansia muciniphila*, *Dysosmobacter welbionis*, *Subdoligranulum variabile*, *Faecalibacterium prausnitzii* and *Christensenella minuta* have been shown to have beneficial effect by countering obesity onset and development (Everard et al., 2013; Goodrich et al., 2014; Munukka et al., 2017; Le Roy et al., 2022). However, correlations of the abundance of specific bacterial species with obesity markers in humans has not always translated into identification of causal relationships. Thus, despite strong negative correlation of variable abundance of *Subdoligranulum* with many obesity traits such as fat mass, adipocyte size, insulin resistance and low-grade inflammation, no impact on metabolic functions was observed in diet-induced obese animal model supplemented by this bacterium (Van Hul et al., 2020).

Initially, several mechanisms centring on a local effect within the gastrointestinal tract have been proposed to explain the effect of microbiota on host metabolic responses. Namely, proposed mechanisms involved suppression of intestinal lipoprotein lipase inhibitor (Angptl4/Fiaf), inactivation of AMP-activated protein kinase (AMPK) pathways, more efficient caloric extraction from complex carbohydrates, effects on intestinal motility and secretion of intestinal hormones (Backhed et al., 2004, 2007; Cani et al., 2008, 2009; Turnbaugh and Gordon, 2009; Turnbaugh et al., 2009; Thaiss et al., 2018). In addition, compromised intestinal integrity is a common feature of obesity and related metabolic diseases and it plays an important role in obesity-related low-grade inflammation. Indeed, gut commensal bacteria appear to be key players in intestinal barrier dysfunction, as antibiotic treatment prevented HFD-induced intestinal permeability (Cani et al., 2008). This is also supported functionally by findings showing that by improving the intestinal barrier functions administration of *A. muciniphila* reduced body weight, adipose tissue inflammation and glucose tolerance in mouse models (Everard et al., 2013; Derrien et al., 2017; Depommier et al., 2019). Subsequently, intestinal permeability was also correlated to increased visceral adiposity and fat accumulation in the liver in humans (Gummesson et al., 2011). The observation of intestinal permeability changes in obese patients and the fact that the intestinal microbiome provides a vast repertoire of different small molecules paved the way for research on the impact of the gut microbiota beyond the gut (Ivanov and Honda, 2012; Donia and Fischbach, 2015; Han et al., 2021). Indeed, it is clear now that microbiota-derived molecules that are absorbed from the gastrointestinal tract can enter the blood circulation via the portal vein and can exert a systemic effect on multiple organs. Obese animals are also characterised by increased permeability of the gut and a subsequent increase in circulating lipopolysaccharide (LPS) (Cani et al., 2008). Landmark studies demonstrate that LPS acts by binding to the Toll-like receptor-4 (TLR4) expressed in various peripheral organs, triggering low-level inflammatory events associated with obesity and metabolic syndrome (Cani et al., 2007; Lu et al., 2008). These pioneering studies emphasized the role of PRRs and their ligands, the MAMPs, on host metabolism.

## PRRs and microbiota derived MAMPs

The innate immunity is an evolutionarily conserved part of the host defence system that forms the first line of defence against pathogens [for review see (Iwasaki and Medzhitov, 2015)]. Innate immunity responses are mediated by a repertoire of germline-encoded pattern recognition receptors (PRRs) that include Toll-like receptors (TLRs), nucleotide-binding oligomerization domain (NOD)-like receptors (NLRs), retinoic acid-inducible gene 1 (RIG-I)-like receptors, absent in melanoma 2 (AIM2)-like receptors, Alpha Kinase-1 (ALPK1) and C-type lectin receptors [(Zhou et al., 2018) and for review see (Iwasaki and Medzhitov, 2015)]. First discovered in the late 1990s, PRRs were described as sensors of invading pathogens through the detection of conserved molecular patterns absent in the host. These patterns were first termed pathogen-associated molecular patterns (PAMPs). Microbial ligands sensed by PRRs are not unique to pathogens but are also expressed by commensal bacteria. The traditional view of innate immune responses as quiescent in the absence of threats and activated only upon pathogen recognition has evolved since the advent of microbiota-centred research. The recent research on human microbiota showed fundamental connections to PRRs leading to the emergence of the term microbe-associated molecular patterns (MAMPs). In addition to MAMPs, some PRRs detect endogenous stress signals through molecules called danger/damage-associated molecular patterns (DAMPs) or alarmins that are released from injured and inflamed tissues or dying cells (Bianchi, 2007).

**Figure 1:**
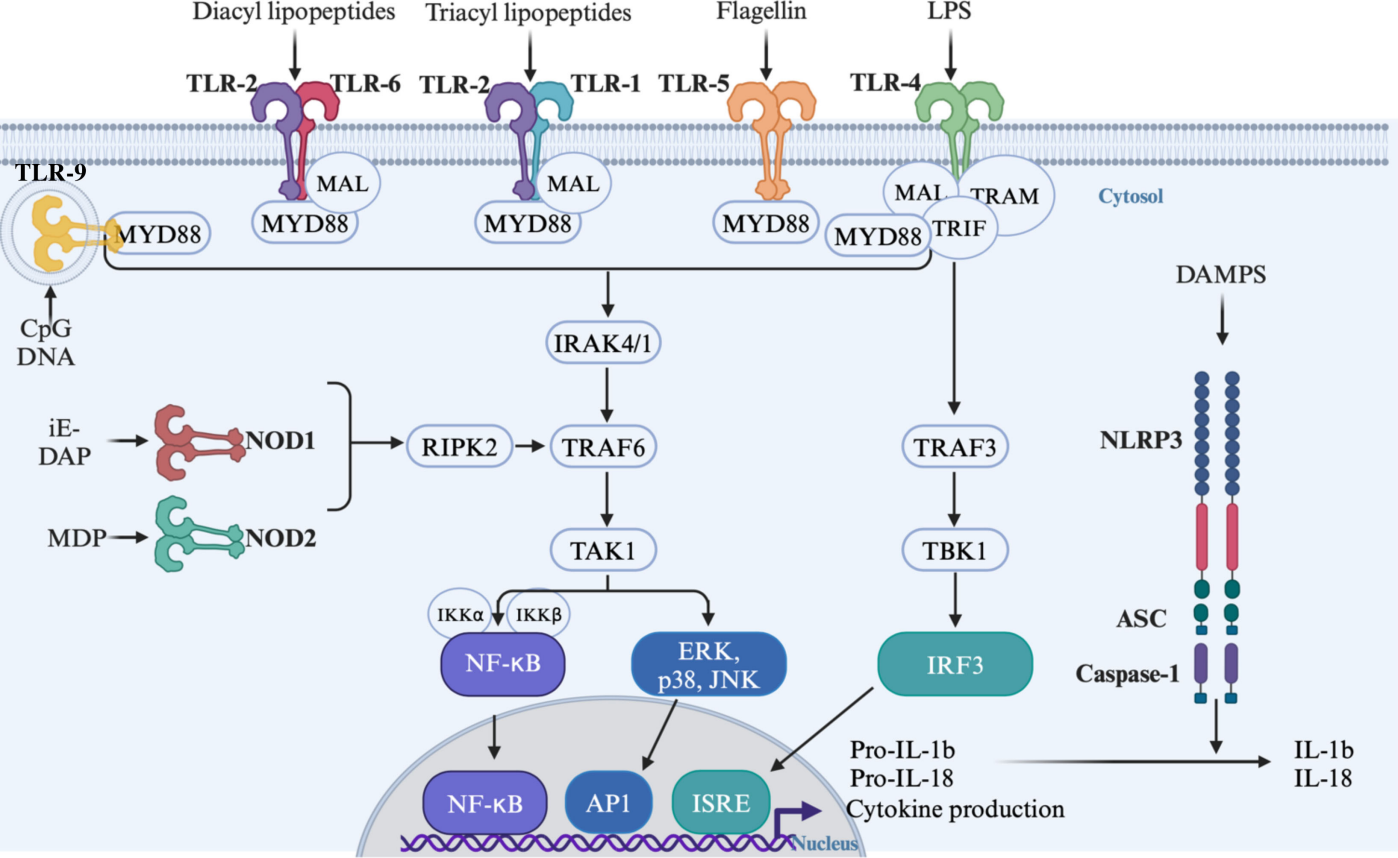
Signalling cascade following activation of TLRs and NODs by their respective ligands. TLR, Toll-like receptors; NOD, Nucleotide oligomerization domain; NLRP3, NOD-like Receptor pyrin domain containing 3; ASC, apoptosis-associated speck-like proteins containing a caspase-recruitment domain; MYD88, Myeloid differentiation primary response 88; MAL, MyD88 adaptor like; TRIF, TIR domain containing adapter-inducing interferon Beta; TRAM, TRIF-related adaptor molecule; IRAK, IL-1 receptor-associated kinases; TRAFs, TNF receptor-associated factors; TAK1, TGF-b-activated kinases 1; TBK1, TANK-binding kinase 1; IKK, Inhibitor of kappa B kinases; RIPK2, Receptor-interacting serine/threonine-protein kinase; NF-κB, Nuclear factor kappa B; ERK, Extracellular signal regulated kinase; JNKs, c-Jun N-terminal kinases; IRF3, Interferon-regulatory factor 3; AP1, activator protein 1; IRSE, IFN- stimulated response elements; IL, interleukin; LPS, Lipopolysaccharide; iE-DAP, y-D-Glu-mDAP; MDP, muramyl dipeptide.

TLRs are transmembrane protein receptors belonging to the Toll/Il1R homology (TIR) domain superfamily. Up to date, thirteen functional TLRs have been reported: ten in humans (TLR1 to TLR10) and twelve in mice (TLR1 to TLR9, TLR11 to TLR13) [for review see (Iwasaki and Medzhitov, 2015)]. TLR1, TLR2, TLR4, TLR5, TLR6 and TLR11 are expressed at the cell surface to detect mainly microbial membrane components whereas TLR3, TLR7, TLR8 and TLR9 are localized in intracellular compartments to sense microbial nucleic acids. These receptors sense a wide range of specific ligands including commensal bacteria-derived molecules resulting in the formation of homodimers at the exception of TLR2, TLR1 and TLR6 that form heterodimers (Fukata and Arditi, 2013). TLR4 recognizes mainly lipopolysaccharide (LPS) derived from Gram-negative bacteria, TLR9 recognizes unmethylated CpG DNA and TLR5 senses flagellin derived from flagellated bacteria (Hoshino et al., 1999; Bauer et al., 2001; Hayashi et al., 2001). TLR2 forms heterodimers with either TLR1 or TLR6 to detect lipopeptides, peptidoglycan, lipoteichoic acid, pilus-like proteins and porins (Takeuchi et al., 2001, 2002). Upon ligand binding, TLRs recruit adaptor proteins such as myeloid differentiation primary response 88 (MyD88), MyD88 adaptor like (MAL), TIR domain-containing adaptor protein (TIRAP), TIR domain containing adapter-inducing interferon beta (TRIF) and TRIF-related adaptor molecule (TRAM) via a TIR-TIR domain interaction [for review see (Luo et al., 2019)]. The recruitment of these adaptors triggers signalling cascades involving several proteins such as IL-1 receptor-associated kinases (IRAKs), TNF receptor-associated factors (TRAFs), TGF-β-activated kinases 1 (TAK1) and inhibitor of kappa B kinases (IKK) leading to the activation of NF-κB, the mitogen-activated protein kinases (MAPKs, e.g. p38 and JNKs) and the transcription factor interferon-regulatory factor 3 (IRF3) (Kawai and Akira, 2007) (**Figure 1**).

NLRs are a large family of cytoplasmic PRRs (22 in humans and 34 in mice) that recognize a wide range of cytosolic MAMPs and DAMPs. Despites the clear role in innate immune responses against pathogens and endogenous damage described for most of them, the functions of an important number of NLRs remain to be explored. The most extensively studied NLRs are two members of the NLRC family: NOD1 and NOD2 which sense peptidoglycan (PGN) found in all bacteria cell wall. NOD1 detects the γ-D-glutamyl-meso-diaminopimelic acid (iE-DAP) expressed in all Gram-negative bacteria and few Gram-positive bacteria. NOD2 recognizes the muramyl dipeptide (MDP) present in all bacteria (Caruso et al., 2014). Similarly to TLRs, NOD1 and NOD2 activation by their ligands induces their oligomerization (Girardin et al., 2001, 2003). The downstream signalling cascade implicating receptor-interacting serine/threonine protein kinase 2 (RIP2), TGF activated kinase 1 (TAK1) and TRAF6 stimulate the IKK/NF-κB complex and MAPK pathway (Caruso et al., 2014) (**Figure 1**). NLRP3 is a member of the NLRP family involved in inflammasome activation. Initially, extracellular ATP and pore-forming toxins were identified as NLRP3 activators (Mariathasan et al., 2006). Subsequent studies have revealed that a wide range of structurally and chemically different molecules can activate NLRP3 without establishing clear direct binding to the receptor (He et al., 2016). The current accepted model involves a two-signal mechanism: i) a priming signal provided by PRR-dependent activation of NF-κB by DAMPS and MAMPS inducing NLRP3, pro-IL-1β, pro-IL-18, apoptosis-associated speck-like proteins containing a caspase-recruitment domain (ASC) and pro-caspase-1 expression; and ii) an activation signal by viral compounds, ATP, bacterial toxins and danger signals such as ionic fluxes, mitochondrial ROS, oxidized mitochondrial DNA and lysosomal damages (Bauernfeind et al., 2009; Elliott and Sutterwala, 2015). Upon activation, NLRP3 oligomerizes and recruits ASC and pro-caspase-1. This complex formation leads to the cleavage-mediated activation of caspase-1 which results in the processing of pro-IL-1, pro-IL18 and Gasdermin D into their active forms (**Figure 1**). This secretion initiates potent inflammatory responses and cell death by pyroptosis (Elliott and Sutterwala, 2015; He et al., 2016).

## Contribution of PRRs to obesity and metabolic diseases

Gut microbiota is composed of a complex variety of microorganisms, especially commensal bacteria, which has co-evolved and established a beneficial symbiotic relationship with the host. In recent years, it has become evident that PRRs constitute key regulators of this host-commensal microbiota interactions which lead to a variety of physiological consequences. PRR-dependent signalling drives the expression of a wide range of genes beyond the inflammatory and immune responses. Indeed, PRRs play a crucial role in the development and the maintenance of intestinal integrity, regulation of local immunity and consequently act as a protective barrier against pathogens. However, in several diseases including obesity and metabolic syndromes, increased gut permeability called “leaky gut” is observed, leading to circulating MAMPs (first described as endotoxemia for LPS) and therefore to the activation of the PRRs in various insulin-responsive tissues. This activation is largely suspected to promote low-grade inflammation in peripheral organs called metaflammation (Cani et al., 2007; Hotamisligil, 2017).

Therefore, the outcomes of the activation of PRR signalling pathways depend on the specific functions of the targeted cells and on their physiological context. This chapter explores the multifaceted role of TLRs, NODs and NLRP3 in obesity-related inflammation, insulin resistance, metabolic dysregulation, and in obesity-related barrier dysfunctions.

### TLR2

TLR2 is expressed on a number of cells from insulin-responsive tissues including macrophages, adipocytes, skeletal muscle cells and hepatocytes (Lin et al., 2000; Lang et al., 2003; Dasu et al., 2010). Along with TLR4, TLR2 expression is increased in adipose tissues, PBMCs and monocytes from obese and diabetic individuals (Creely et al., 2007; Dasu et al., 2010; Poulain-Godefroy et al., 2010; Ahmad et al., 2012) with a significant correlation with body mass index (BMI). Overexpression of TLR2, but not TLR4, is associated with low-grade chronic inflammation in individuals with metabolic healthy obesity (individuals with a BMI>30 but displaying no metabolic abnormalities such as insulin resistance, hypertension and dyslipidemia) (Guerrero-Romero et al., 2023). Elevated expression of TLR2 in epididymal fat tissue was also confirmed in high-fat Diet (HFD containing 60% of total calories from lipid) fed-animals (Davis et al., 2011) for 16 weeks and in white adipose tissue and liver of ob/ob mice (Kuo et al., 2011). The increased expression of TLR2 suggested an increased activation of inflammatory signalling in obesogenic context. Despites some controversies, most studies using global deficiency of TLR2 suggested a potential role of this PRR in diet induced obese animal models (Ehses et al., 2010; Himes and Smith, 2010; Davis et al., 2011; Kuo et al., 2011; Guo et al., 2021). TLR2 deficiency on HFD-induced obesity background ameliorates tissue inflammation (liver and adipocyte tissue), macrophage infiltration, insulin sensitivity (in the liver, muscle and adipose tissue), glucose tolerance and weight gain, and reduces adiposity despite a normal energy intake and absorption when compared to WT mice (Himes and Smith, 2010; Davis et al., 2011; Guo et al., 2021). Interestingly, a study showed that locomotor activity and consequently energy expenditure were increased in HFD-fed TLR2^-/-^ mice. Altogether, these studies point to a pathogenic role of TLR2 in obesity and insulin resistance via induction of inflammation in insulin-responsive tissues. In contrast to the above-described studies, a work reported that TLR2 deficiency in aged mice exacerbated HFD-induced obesity. This phenotype was explained by an increased TLR2 expression in aged mice, an increased food intake and reduced levels of the hypothalamic anorexigenic peptide α-melanocyte-stimulating hormone in TLR2^-/-^ (Shechter et al., 2013). TLR2 is also involved in the mechanism of action underlying improvement of gut barrier function triggered by the pili-related protein Amuc_1100. This protein is secreted by *A. muciniphila* and interacts and activates TLR2 (Plovier et al., 2017). Purified Amuc_1100 administration increased the expression of the tight-junction genes, *Occludin* and *Claudin 3* and decreased the endotoxemia in pre-clinical models. Activation of TLR2 by Amuc_1100 could partially recapitulate the beneficial effect of *A. muciniphila* on host metabolism in both obese and diabetic models (Plovier et al., 2017; Song et al., 2023). Interestingly, the impact of TLR2 as an important regulator of tight junction gene expression and epithelial barrier integrity has been reported elsewhere in non-obese context (Cario et al., 2007).

TLR2, via its activation by molecules derived from the microbiota clearly plays a role in the onset of obesity. Presumably, this receptor can trigger the organ-specific inflammation, the reinforcement of the intestinal barrier or the induction of anorexigenic signals in the hypothalamus that differentially affect the systemic metabolism. Cell-specific deletion of TLR2 in mice models are thus needed to clearly establish the detailed cell- or organ-specific role of this receptor.

### TLR4

TLR4 binds not only LPS of Gram-negative bacterial cell walls (Calabrese et al., 2015), resistin (Tarkowski et al., 2010) and alarmin (Harris et al., 2012). While previous studies mentioned free fatty acids (FFA) as potential ligands of TLR4 (Shi et al., 2006; Rocha et al., 2016), it was recently clearly established that FFA do not bind or activate directly TLR4 (Lancaster et al., 2018). However, TLR4 is necessary for FFA to trigger its pro-inflammatory effects (Lancaster et al., 2018). Obese human subjects and diet-induced mouse models of obesity display higher circulating level of LPS that can all initiate an immune response through their interactions with TLR4. TLR4 is highly expressed in the innate immune cells, but also in non-immune cells of tissues involved in the onset metabolic syndrome including endothelial cells (Hijiya et al., 2002), pancreatic β-cells (Garay-Malpartida et al., 2011), adipocytes (Vitseva et al., 2008), neurons (Buchanan et al., 2010) and hepatocytes (Matsumura et al., 2000; Migita et al., 2004; Jia et al., 2014). Several studies have shown that global TLR4-deficient mice are protected against HFD-induced insulin resistance and inflammation associated with a modest or no reduction of body weight gain (Shi et al., 2006; Kim et al., 2007; Suganami et al., 2007; Radin et al., 2008). Furthermore, the implication of specific TLR4-expressing cell types contributing to HFD-diet induced metabolic disorders was investigated. When fed HFD, mice lacking TLR4 specifically in hepatocytes have shown improved glucose tolerance, enhanced insulin sensitivity, reduced hepatic steatosis and a significant decrease in tissue and circulating inflammatory markers despite undergoing similar body weight gain as WT mice (Jia et al., 2014). Chronic low-grade inflammation in adipose tissue is a major modulator of obesity-induced insulin resistance, therefore, the role of TLR4 in adipose tissue inflammation and development has been extensively investigated. TLR4 is expressed in adipocytes in mice (Shi et al., 2006) and humans (Bruno et al., 2021). *In vivo*, TLR4 mRNA expression was increased in epididymal adipose tissues of obese (*ob/ob* and diet induced obesity) and diabetic (*db/db*) mice models (Shi et al., 2006). *In vitro*, TLR4 expression increases during 3T3 adipocyte differentiation, and its silencing prevents this differentiation (Schaeffler et al., 2009). In accordance to these data, several studies reported that TLR4^-/-^ mice displayed reduced fat pad expansion in response to long-term exposure to HFD. TLR4 is also upregulated in 3T3 adipocytes in response to FFAs exposure (Schaeffler et al., 2009) and specific deletion of TLR4 in adipocytes prevents FFA-induced inflammation *in vitro* and reduces adipose tissue inflammation in mice model of diet induced obesity (Shi et al., 2006). In white adipose tissue, TLR4 is also expressed in resident macrophages (Nguyen et al., 2007). ATM are surrounded by adipocytes that constantly release FFAs that have the potential to activate macrophages. However, the mechanism recently identified by Lancaster indicates that FFA-induced inflammation does not result from the directed activation of TLR4 by FFA (Lancaster et al., 2018). It has been suggested that TLR4-expressing immune cells, especially macrophages, are involved in regulating obesity-induced metabolic disorders. However, their role remained unclear: TLR4 ablation in hematopoietic cells showed conflicting results in protecting mice from obesity-induced insulin resistance (Saberi et al., 2009; Orr et al., 2012) and TLR4 ablation in myeloid cells does not ameliorate HFD-induced insulin resistance (Jia et al., 2014). However, global deletion of TLR4 dampens inflammation in white adipose tissue and promotes macrophages polarization toward M2 phenotype (Orr et al., 2012). In pancreas, TLR4 expression also increases in response to LPS and alters β-cell viability (Goldberg et al., 2007; Garay-Malpartida et al., 2011) and insulin secretion (Garay-Malpartida et al., 2011) suggesting that TLR4 may also control glucose homeostasis by regulating directly insulin production by the β-cells and not only through its role as a trigger of inflammation and insulin resistance in peripheral tissues. In addition to the role of the TLR4 expressing peripheral tissues, the development of insulin resistance induced by lipid consumption is also mediated in part by activation of the TLR4 inflammatory pathway in the hypothalamus (Milanski et al., 2009).

Altogether, *in vitro*, rodent preclinical models of obesity and human studies emphasized the central role of TLR4 activation in various tissues as a key player that connects HFD with metabolic inflammation and insulin resistance.

### TLR5

TLR5 is strongly expressed in numerous types of epithelial cells in various organs including the gastrointestinal tract, where it is present on the basolateral side of the colonic epithelial cells, and the intestinal CD11c(+) cells of the lamina propria of the small intestine (Gewirtz et al., 2001; Uematsu et al., 2006). TLR5 is also present in human adipose tissues (Pekkala et al., 2015), in the resident macrophages of murine and human pancreatic islets (Scheithauer et al., 2022) as well as in the liver, spleen and lungs (Etienne-Mesmin et al., 2016). TLR5 recognizes an evolutionarily conserved 13-amino acid motif present on flagellin of bacteria flagella. Upon its activation by flagellin TLR5 initiates NF-κB signalling pathway activation in a MyD88- or TRIF- dependent manner, resulting in proinflammatory cytokine production (IL1, IL6, and TNFα) (Gewirtz et al., 2001; Choi et al., 2010). Recently, faecal flagellin levels have been reported to be higher in overweight compared to normal weight human subjects and even higher in obese subjects (Tran et al., 2019). Similar increase in flagellin levels was observed in diet-induced obese mice compared to lean counterparts (Tran et al., 2019; Scheithauer et al., 2022) suggesting a strong association between the main TLR5 ligand and body weight. These studies supported original findings made using TLR5^-/-^ mice (Vijay-Kumar et al., 2010). When fed normal diet, adult male and female TLR5^-/-^ mice gained more weight, displayed higher adiposity, strong dyslipidemia, elevated levels of proinflammatory cytokines in the gut and adipose tissue, impaired regulation of glycemia and strong insulinemia when compared to their WT littermates (Vijay-Kumar et al., 2010). This phenotype was similar in mice that lacked TLR5 solely in IEC suggesting a key role of intestinal inflammation in TLR5^-/–^dependent metabolic impairment (Chassaing et al., 2014). Under HFD feeding, metabolic syndrome features were exacerbated in TLR5^-/-^ mice when compared to WT littermates. Specifically, HFD-fed TLR5^-/-^ mice displayed a strong hyperinsulinemia and insulin resistance (Vijay-Kumar et al., 2010). TLR5^-/-^ mice also developed gut microbiota dysbiosis, that when transferred to WT mice via faecal transplant recapitulated the characteristics of metabolic syndrome. This suggests a key role of the gut microbiota in driving TLR5^-/–^dependent metabolic disorders (Vijay-Kumar et al., 2010). However, other studies also reported that changes in microbiota composition are not solely the result of TLR5 deficiency: the environment in which TLR5^-/-^ mice are hosted can affect microbiota adaptation to TLR5 deletion and possibly the TLR5-dependent metabolic syndrome onset (Zhang et al., 2016; Lipinski et al., 2021). Among obese subjects, faecal flagellin gene abundance was shown to be higher in individuals with T2D than in those with normal glycemia (Scheithauer et al., 2022). Furthermore, serum flagellin levels correlated positively with HbA1c in T2D obese subjects (Scheithauer et al., 2022) emphasizing the role of flagellin in the dysregulation of glucose metabolism. Flagellin can reach the circulation after a meal in human obese subjects suggesting that systemic gut derived flagellin could directly stimulate immune response in TLR5 extra-intestinal tissue such as pancreatic islet, liver or adipose tissues. In pancreas, Scheithauer and collaborators demonstrated that circulating flagellin activates TLR5 expressed in resident islet macrophages and consequently promotes pro-inflammatory state in beta cells, impairs insulin gene expression and proinsulin processing leading to reduced insulin storage and hyperinsulinemia *in vivo* in a TLR5-dependent manner (Scheithauer et al., 2022). To the opposite, in the liver, like in intestine, TLR5 deletion seems to be protective. Indeed, mice lacking TLR5 specifically in hepatocytes (TLR5^ΔHep^) were more prone to liver steatosis and liver inflammation when exposed to HFD than their WT littermates and this effect was microbiota-dependent since antibiotic treatment reduced hepatic steatosis and fibrosis in HFD-TLR5^ΔHep^ mice (Etienne-Mesmin et al., 2016). In the adipose tissue, the role of TLR5 is less clear. Whole body TLR5 deletion is associated with an increase in adiposity and adipose tissue inflammation likely as consequence of gut barrier defect and gut microbiota dysbiosis (Vijay-Kumar et al., 2010). However, humans with high expression levels of TLR5 signalling pathway genes displayed a higher fat mass and more pronounced metabolic alterations than subjects with lower expression of TLR5 signalling pathway related genes (Pekkala et al., 2015). Flagellin also impairs insulin-signalling pathway and inflammation in human adipocytes cultured *in vitro* suggesting a deleterious effect of TLR5 activation on adipocytes functions (Pekkala et al., 2015). In line with these finding, nonsense polymorphism in TLR5, equivalent to loss of TLR5 function, has been associated with protection from obesity and lower BMI in human (Al-Daghri et al., 2013).

In conclusion, obesity and related metabolic diseases, including T2D, are clearly associated with TLR5-dependent immune response. Further human studies with larger cohorts are needed to confirm the protective effect of TLR5 deletion to obesity as observed in mice. However, in extra-intestinal tissue such as pancreatic islets and possibly in adipocyte, TLR5 also promotes inflammation (Scheithauer et al., 2022).

### TLR9

TLR9 is an intracellular receptor which recognizes bacterial non-methylated DNA containing CpG motifs (Wagner, 2001), viral double-stranded DNA (Krug et al., 2004) as well as cell-free nucleic acids originating from damaged cells (Kumagai et al., 2008). TLR9 is expressed in the endoplasmic reticulum of various immune cell types, including macrophages, B cells, dendritic cells, and plasma cells (Leifer et al., 2004). After activation, TLR9 signals through MyD88 and leads to induction of NF-κB-dependent inflammatory cytokine secretion (Kawai and Akira, 2011). TLR9 has been shown to be involved in obesity development and related blood glucose alteration (Hong et al., 2015). TLR9 receptor is expressed in murine and human adipose tissues (Thomalla et al., 2019). Its expression is induced in 3T3-L1 adipocytes during their differentiation (Thomalla et al., 2019) and in murine adipose tissue in response to HFD (Nishimoto et al., 2016). Moreover, deletion of TLR9 prevents inflammation and insulin resistance in HFD-induced obesity mice model (Nishimoto et al., 2016; Revelo et al., 2016). In obese individuals, expression of TRL9 is higher in visceral than in subcutaneous fat tissue and correlates positively with the circulating levels of resistin, a pro-inflammatory adipokine suspected to be an important link between obesity and insulin resistance (Thomalla et al., 2019). However, up to now it is mostly the cell-free DNA originating from apoptotic adipocytes that has been implicated as the TLR9 ligand that induces a TLR9-dependent pro-inflammatory response in adipose tissue in the context of obesity (Nishimoto et al., 2016; Revelo et al., 2016). So far, there is no evidence that bacterial DNA contributes to the TLR9-dependent metabolic inflammation in obesity even though, disturbed gut microbiome composition and increased intestinal permeability are usually associated with murine and human obesity and could potentially contribute to increased circulating levels of bacterial DNA fragments. Further research is still needed to clarify this possibility.

### NOD1

NOD1 is ubiquitously expressed in various tissues and cells (Inohara et al., 1999) and particularly in immune cells (Clarke et al., 2010) and adipocytes (Zhou et al., 2015). Activation of NOD1 by its ligand (iE-DAP) leads to the activation of the NF-κB pathway (Chamaillard et al., 2003). As shown for TLR4 and TLR2, NOD1 expression is upregulated in the adipose tissue of obese mice (Fernandez-Garcia et al., 2021) and obese individuals with metabolic syndrome (Zhou et al., 2015). *In vitro*, incubation of 3T3 adipocytes with NOD1 ligand induced release of pro-inflammatory cytokines (MCP1, TNFα and IL-6), altered insulin signalling and decreased glucose uptake (Zhao et al., 2011). NOD1expression and activation also positively correlate with insulin resistance in obese humans (Zhou et al., 2015). Intraperitoneal injection of NOD1 ligand in lean mice decreases insulin sensitivity which can be abolished by NOD1 deletion (Schertzer et al., 2011) confirming that activation of NOD1 impairs glucose metabolism. Like LPS, obesogenic diet consumption leads to elevated plasmatic levels of NOD1 ligand as the result of impaired intestinal permeability and translocation of bacteria and bacterial fragments from the gut into the systemic circulation (Chan et al., 2017). Rodent models with either a global or partial NOD1 deficiency provided a better understanding of the role of NOD1 receptors on the development of obesity. HFD-fed NOD1^-/-^ mice did not gain weight and had comparable adiposity as well as glucose tolerance after one month of HFD compared to WT mice (Amar et al., 2011). NOD1 deficiency in immune cells is sufficient to enhance insulin sensitivity independently of change in body weight and adiposity (Chan et al., 2017). Silencing NOD1 in immune cells also reduces M1 macrophage polarization in epididymal adipose tissue of HFD-fed mice compared to WT despite a similar density of total infiltrated macrophages (Chan et al., 2017) suggesting that NOD1 expressing immune cells are major contributors to metabolic inflammation and insulin resistance in response to HFD. Unlike the studies described above, one study described that absence of NOD1 enhances body weight gain and adiposity in mice fed HFD for 6 weeks when compared to WT (Gonzalez-Ramos et al., 2020). In the study, the authors suggested that HFD-fed NOD1^-/-^ mice had a lower basal metabolic rate as the result of altered thyroid function. However, the differences in metabolic outcomes described in this study in response to NOD1 deletion could also be explained by the different genetic background of the mice. The role of NOD1 in blood glucose regulation has also been studied in humans in association with obesity. Elevated NOD1 expression in monocytes was positively correlated with insulin resistance and waist circumference in Indian human cohort (Shiny et al., 2013) and increased NOD1 expression in omental adipose tissue was associated positively with gestational diabetes (Lappas, 2014). Genetic studies showed that the polymorphism rs2075820 on NOD1 locus modifies the association between dietary lipids and insulin sensitivity in healthy individuals but is not associated with any biomarkers of the metabolic syndrome (Cuda et al., 2012; Ozbayer et al., 2017). Taken together, these findings highlight the importance of NOD1 in the development of obesity-associated glucose regulation impairment and of the role of NOD1 expressing immune cells in this effect.

### NOD2

NOD2 is a cytoplasmic receptor of innate immunity that is mainly expressed in immune cell (specifically monocytes and macrophages) (Inohara and Nunez, 2003). It is activated by muramyl dipeptide (MDP), which is ubiquitously expressed in all bacteria (Girardin et al., 2003). Its activation leads to the secretion of IL-1β, TNFα, IL-6, IL-12p40, CC-chemokine ligand 2 (CCL2), the neutrophil chemoattractants CXC-chemokine ligand 8 (CXCL8, also known as IL-8 in human) and CXC-chemokine ligand 2 (CXCL2, also known as MIP-2a) and is involved in neutrophils, monocytes and dendritic cells recruitment (Negroni et al., 2018). Beyond its role in innate immunity, NOD2 appears to protect against obesity and associated metabolic diseases. *In vitro*, MDP reduces myotube insulin sensitivity (Tamrakar et al., 2010). *In vivo*, intraperitoneal injection of HFD-fed obese mice with MDP improves their insulin sensitivity, glucose tolerance and lowers their hepatic glucose production (Cavallari et al., 2017). NOD2 deficiency in both BALB/c mice (known to be resistant to diet-induced obesity) and HFD-fed C57BL/6 mice leads to severe weight gain and increased adiposity, altered glucose regulation and insulin sensitivity as well as changes in adipose tissue and hepatic transcriptomic profiles (Denou et al., 2015; Rodriguez-Nunez et al., 2017; Carlos et al., 2020). Conversely, MDP injection in obese mice lacking NOD2 in hepatocytes (Cavallari et al., 2017) or specific deletion of NOD2 in hematopoietic-derived immune cells in HFD-fed mice (Denou et al., 2015) did not restore or impaired glucose homeostasis, respectively, suggesting a limited role of the liver and immune cells in the protective effect of NOD2 activation on glycemia. Irradiated mice that were reconstituted by bone marrow transplantation from RIPK2^-/-^ (an intermediate kinase central in NOD1/2 signalling pathway) mice displayed normal glucose homeostasis after MDP injection, confirming that NOD2 activation in immune cells is not required for glucose regulation (Cavallari et al., 2020). Transfer of the microbiota from HFD-fed NOD2^-/-^ mice recapitulates part of the inflammation and glucose homeostasis effects seen in HFD-fed NOD2^-/-^mice (Denou et al., 2015). NOD2 is also involved in energy homeostasis by modulating food intake and body temperature (Gabanyi et al., 2022). Indeed, NOD2 is expressed in the hypothalamus and MDP can modulate the firing activity of specific hypothalamic neurons. In female mice, NOD2 deficiency in GABAergic neurons suppresses appetite control and delays satiety leading to increase in body weight gain. To the opposite, administration of MDP by gavage to WT mice decreases food intake suggesting that MDP acts as a satiety signal. In addition, mice lacking NOD2 in GABAergic neurons have difficulty adjusting their body temperature to environmental changes suggesting that central regulation of thermogenesis could also be involved in the metabolic outcome of NOD2 deficiency (Gabanyi et al., 2022).

In humans, expression of NOD2 along with NOD1 increases in monocytes from individuals with type 2 diabetes (Shiny et al., 2013). However, human genetic studies did not show any association between known NOD2 polymorphisms (respectively rs2066842 and rs2066847) and type 2 diabetes (Cuda et al., 2012; Ozbayer et al., 2017).

Altogether, these studies emphasized the importance of NOD2 activity in obesity context: while NOD2 deficiency promotes obesity-induced metabolic dysfunction, activation of NOD2 by its ligand improves glucose tolerance and insulin resistance.

### NLRP3

NLRP3 is a cytosolic protein involved in inflammatory processes and pyroptosis-dependent cell death (Elliott and Sutterwala, 2015; He et al., 2016). The NLRP3 inflammasome involves a two-way signals leading to the maturation and release of the IL-1β and IL-18, two inflammatory cytokines implicated in metabolic inflammation and impairment of insulin signalling (Jager et al., 2007; Elliott and Sutterwala, 2015; He et al., 2016).

A decade ago, pioneering studies demonstrated that mice deficient in NLRP3 are protected from HFD-induced obesity, glucose sensitivity and insulin resistance (Stienstra et al., 2011; Vandanmagsar et al., 2011; Wen et al., 2011). These observations were also confirmed in mice deficient for other protein members of the NLRP3 inflammasome such as Asc and Caspase-1 (Stienstra et al., 2010, 2011; Wen et al., 2011). This protective phenotype was associated with reduced adipocyte hypertrophy and reduced infiltration of macrophages in the visceral adipose tissues (Stienstra et al., 2011; Vandanmagsar et al., 2011). Two studies established a role of macrophages in the NLRP3-dependent insulin resistance by activating T cells in adipose tissues or hepatocytes in the liver in TNF- and IL-1β-dependent activation of the Akt pathway (Vandanmagsar et al., 2011; Wen et al., 2011). NLRP3 activation has been also associated with adipocyte homeostasis (Wang et al., 2017). Genetic deletion studies showed that NLRP3 and Caspase-1 activation modulate adipocyte functions and adipocyte differentiation (Stienstra et al., 2010, 2011). The NLRP3 inflammasome induces the expression of pro-adipogenic genes and adipocyte differentiation, notably by IL-1β (Stienstra et al., 2010). In addition, preadipocytes derived from NLRP3 inflammasome-deficient mice display higher insulin sensitivity and higher fat oxidation rates (Stienstra et al., 2010). Furthermore, these studies point out that obesity itself induces and increased expression of NLRP3, Caspase-1 and IL-1β in visceral adipose tissues (Stienstra et al., 2011; Vandanmagsar et al., 2011; Wen et al., 2011). These observations can be paralleled to humans. Indeed, NLRP3 expression is increased in adipose tissues from obese patients compared to lean controls (Esser et al., 2013; Yin et al., 2014; Bando et al., 2015; Kursawe et al., 2016). NLRP3 expression correlated with insulin resistance values and metabolic status of the obese patients (Esser et al., 2013). Interestingly, Vandanmagsar et al. reported that a reduction of NLRP3 and IL-1β expression in adipose tissues, inflammatory markers and improved insulin sensitivity were associated with weight loss in obese type-2 diabetes patients (Vandanmagsar et al., 2011).

Several DAMPs have been reported to be involved in NLRP3 activation and IL-1β maturation in HFD-induced obesity models. Ceramides, saturated fatty acids such as palmitate, and islet amyloid polypeptide (IAPP) are potent activators of the NLRP3 inflammasome (Masters et al., 2010; Vandanmagsar et al., 2011; Wen et al., 2011). Palmitate-dependent activation of NLRP3 is linked to autophagy and mitochondria-derived ROS production leading to Il-1β-mediated insulin resistance (Wen et al., 2011). However, how microbiota is link to NLRP3-dependent obesity, glucose sensitivity and insulin resistance remains unclear. As mentioned, obesity is associated with increased gut permeability and a microbiota switch towards Bacteroidetes that produce LPS. These characteristics lead to increased circulating bacterial components such as LPS, which can activate PRR signalling and provide the priming signal for the NLRP3 inflammasome (Cani et al., 2007). Supporting this hypothesis, it was shown that bone marrow–derived macrophages (BMDMs) and adipose tissue explants primed with LPS and then stimulated by ceramides express Caspase-1 and IL-1β in a NLRP3-dependent manner (vander. Altogether, these studies show that the NLRP3 inflammasome plays a key detrimental role in the activation of chronic inflammation, which consequently impacts insulin sensitivity during obesity. The gut microbiota, as a source of MAMPs that trigger innate immune responses and the primary signal for the inflammasome, seems to be involved in this process.

## Conclusion

**Figure 2:**
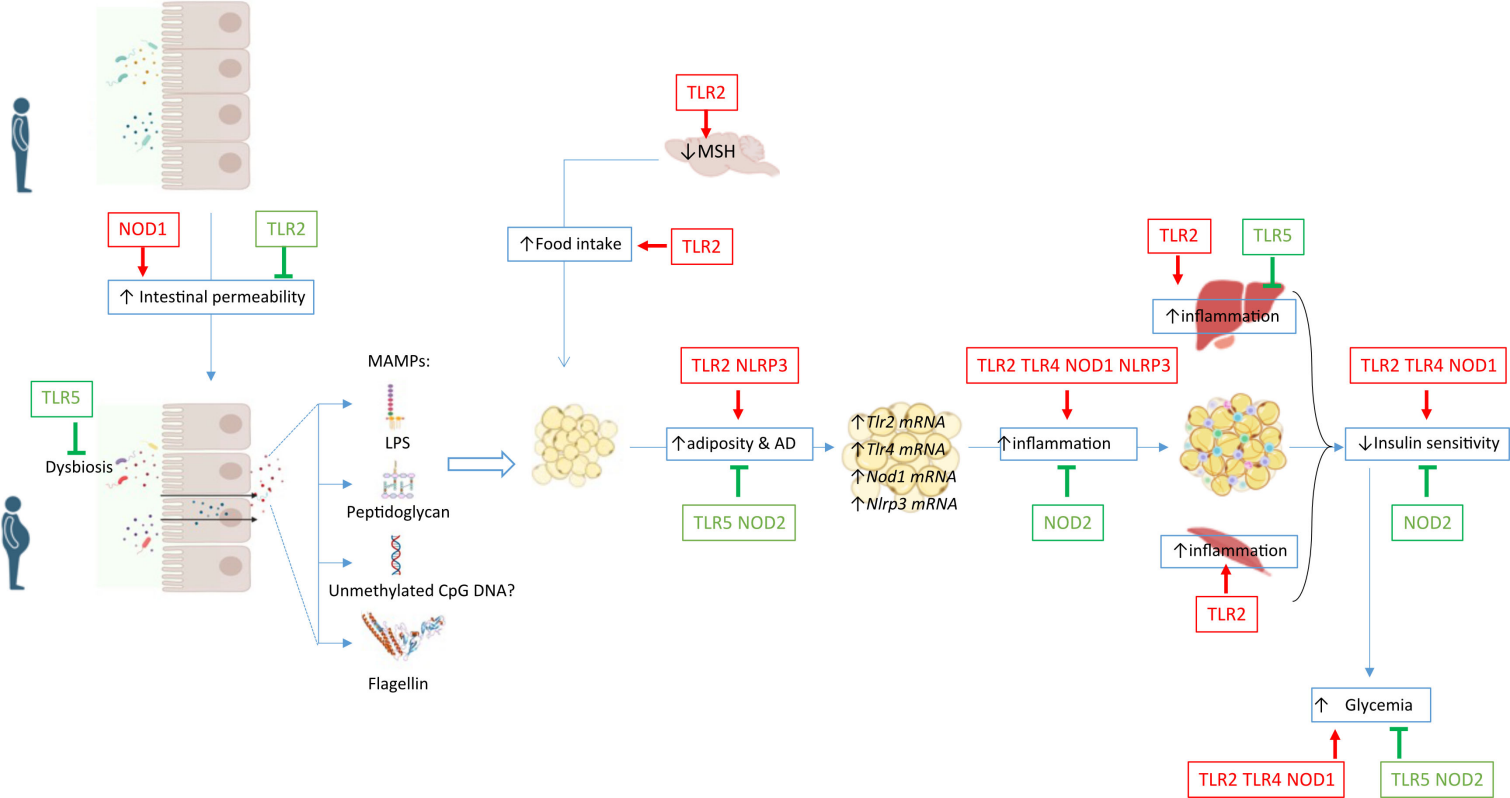
Gut microbiota composition and the intestinal permeability are altered during obesity leading to increased circulating levels of ligands named MAMPs (i.e LPS, peptidoglycan, unmethylated CpG DNA and flagellin) for the PRRs expressed in various metabolic organs. PRRs activation can regulated host metabolic inflammation by altering inflammation and functions of various organs.

Accumulating evidence has now clearly demonstrated the crucial role of the microbiota sensed by PRRs in the host metabolic responses. Activation of PRRs by molecules derived from the microbiota can lead to multiple biological outcomes: reinforcement of the barrier functions of the intestinal epithelium, metaflammation in insulin responsive organs that leads to peripheral insulin resistance and modulation of the neuronal control of energy balance (**Figure 2**). Activations of PRRs by their microbial ligands have multiple impact on the host. The involvement of multiple organs and their diverse physiological functions in the regulation of metabolic homeostasis and the fact that diverse cells express a wide range of PRRs are challenging to clearly decipher the mechanisms involved in obesity and diabetes outcome. Most studies used whole-body genetic deletion models and thus did not clarify the relative contribution of individual PRRs in different tissues and in specific cell types to the development of obesity and metabolic diseases. Further studies using conditional knockout animals and virus-induced local genetic deletion models are needed to decipher the specific role of PRRs in the different organs and cell-types impacting obesity and metabolic diseases. Moreover, the current models cannot decipher the role of PRR in the maintenance or reduction of obesity. Deletion on already obese murine models would answer this remaining question.

In addition to the above discussed mechanisms, the gut microbiota metabolizes dietary nutrients into a myriad of bioactive compounds. They include tryptophan-derived and choline-derived molecules and short chain fatty acids (SCFA) that can regulate the intestinal barrier, host energy utilization, inflammatory response and host metabolism with beneficial or detrimental effects on health (Martin-Gallausiaux et al., 2020; Agus et al., 2021). Thus, the identification of the mechanisms responsible for the metabolic phenotype observed *in vivo* can be further complicated by differences in the gut microbiota composition, which are modulated by long-term dietary intervention and PRR genetic deletion (Denou et al., 2015). Indeed, microbiota composition was not assessed in most of the studies described above. Systematic microbiota composition assessment, short-term dietary intervention, induced gene deletion (tamoxifen induction) and, germ-free and faecal transplant mice models might be used to clearly decipher the precise role of the activation of PRRs by the microbiota in metabolic phenotypes.

In addition to the metabolic consequences of obesity discussed in this review, the systemic inflammation also contributes to the pathogenesis of other inflammatory diseases and cancers (Nobs et al., 2020). Thus, understanding the complex crosstalk between the microbiota and the PRRs, and how this relationship modulates metabolic functions and systemic inflammation could result in advances toward the treatment not only of metabolic diseases but also other inflammatory-related diseases.

## Author contributions

AR: Writing – original draft, Writing – review & editing. VD: Conceptualization, Writing – original draft, Writing – review & editing. NL: Conceptualization, Writing – original draft, Writing – review & editing.
